# Association of Inflammation and Immune Cell Infiltration with Estrogen Receptor Alpha in an Estrogen and Ionizing Radiation-Induced Breast Cancer Model

**DOI:** 10.3390/ijms25168604

**Published:** 2024-08-07

**Authors:** Tania Koning, Gloria M. Calaf

**Affiliations:** Instituto de Alta Investigación, Universidad de Tarapacá, Arica 1000000, Chile; tkoning@academicos.uta.cl

**Keywords:** breast cancer, radiation, estrogen, inflammation, chemokines, interleukins

## Abstract

Breast cancer is the most diagnosed cancer in the world, and it is the primary cause of cancer death for women. The risk of breast cancer is increased by endogenous factors like hormones and exogenous factors like radiation exposure that causes damage to the mammary epithelial cells leading to an inflammatory response. Chronic inflammation creates a microenvironment composed of, among other factors, chemokines, and interleukins, which promote cancer. The gene expression of the interleukin 1 receptor type 1, the interleukin 1 receptor antagonist, the Interleukin 1 Receptor Accessory Protein, the interleukin 6 cytokine family signal transducer, the C-X-C motif chemokine ligand 3, the C-X-C motif chemokine ligand 5, and the C-X-C motif chemokine ligand 6 were analyzed in an estrogen and radiation experimental breast cancer model. Furthermore, the expression of these genes was correlated with immune cell infiltration, estrogen receptor expression, and their clinical relevance in breast cancer patients based on data provided by The Cancer Genome Atlas database online. Results given by the experimental breast cancer model showed that all genes related to inflammation respond to ionizing radiation alone or in combination with estrogen. On the other hand, the immune response depended on the breast cancer type and on the expression of the gene that encoded the estrogen receptor. Finally, the importance of the expression of these genes in breast cancer is such that high *IL1R1* or *IL1RAP* is strongly related to patient survival. These findings may help to improve the understanding of the role of immune molecules in carcinogenesis and enhance therapeutic approaches.

## 1. Introduction

Breast cancer is a major global health concern because it is the most diagnosed cancer worldwide, accounting for 2.26 million cases in 2020, and it is the primary cause of cancer death in women [1]. Despite being traditionally associated with developed nations, over half of all mammary cancer diagnoses and two-thirds of disease-related deaths in 2020 occurred in less developed countries [2].

Breast cancer is a diverse range of illnesses with various pathologies and clinical behaviors, rather than a single illness [3,4,5,6]. The risk of breast cancer increases because of endogenous factors such as hormones and exogenous factors such as exposure to radiation or chemicals in the environment [7]. Within the hormonal background associated with this type of cancer, about 75% of patients suffering from this disease are clinically diagnosed with estrogen receptor-positive (ER+) tumors; thus, in both basic and clinical research, regulating ER expression becomes pivotal [8]. Based on the expression of molecular markers (including ER expression), there are five main molecular subtypes such as luminal A, luminal B, HER2+, basal-like, and normal breast-like [4,5,6]. Different subtypes of tumors have distinct clinical histories and therapeutic outcomes. ER-negative (ER−) cancer patients cannot be treated by endocrine therapy [9].

Radiation is another important factor in cancer biology [7]. Ionizing radiation is a well-established human carcinogen and induces breast cancer. This is characterized by randomly permeating various tissues, hitting different cells, and causing damage according to the dose received [10]. Such damage could change and destroy DNA, RNA, and cell membrane components such as lipids and proteins by direct ionization or by water radiolysis and lead to mutations [11]. The latter includes many reactive oxygen species, which are the primary cause of tissue damage and cell death [10,12]. Physical carcinogens such as radiation have been used as initiators and promoters in cell models of human and animal cell lines [7,13]; however, the underlying cellular and molecular mechanisms of radiation carcinogenesis remain unknown.

Mammary gland cancer typically begins in the epithelial cells of the ducts, where an accumulation of mutational alterations can occur generating cellular damage. Repair of damaged tissue generates an inflammatory response that causes quantitative and qualitative changes in the immune cell population at the site of injured tissue. When inflammation becomes chronic, it can lead to cell mutation and proliferation, often creating a favorable microenvironment for cancer development [14]. This microenvironment is formed by cells such as immune cells, adipocytes, fibroblasts, microbiome, and factors soluble, including growth factors, cytokines, chemokines, and prostaglandins

The interleukin 1 (IL-1) family, long recognized for its pleiotropic effects on inflammation, plays a complex, sometimes contradictory role in different stages of cancer development, there are numerous ligands and receptors in this family; however, IL-1α and IL-1β are the two main agonists [15,16]. IL1R1 is the main receptor for both ligands, and it is expressed by a broad spectrum of cell types, including fibroblasts, adipocytes, chondrocytes, innate and adaptive immune cell types, and epithelial and endothelial cells [17,18]. IL1R1 forms a tertiary complex with the IL-1 receptor accessory protein (IL1RaP, also called IL1R3), facilitating positive signaling, and recruiting downstream cell signaling proteins; however, IL1R1 has an antagonistic ligand IL1RN, also known as IL1RA, that is overexpressed in several cancers, including multiple myeloma, leukemia, cervical, ovarian, colorectal, pancreatic, and breast cancer [17,18,19,20,21,22], but it is downregulated in others [23]. Similarly, high expression of the IL1RN correlates with either better [18,21,24,25,26,27] or worse cancer prognosis [26,28,29].

The Interleukin 6 (IL6)-like family is a group of proteins with similar structure and functional features, and IL6 signal transducer (IL6ST, which is also its gene name) is a transmembrane signaling receptor that acts as a signal transducer in all signaling by this cytokine family [30,31]. IL6ST has been shown to play important roles in homeostasis, immunity, inflammation, and disease pathogenesis, having an established part in numerous cancer types [32], such as breast neoplasms, and such roles are involved in many hallmarks of cancer development and progression [33,34,35].

Chemokines were initially defined as soluble factors regulating the directional migration of leukocytes during states of inflammation; however, chemokine biology extends to all cell types, including most human neoplastic cells [36]. Chemokines emerged as potential factors involved in breast carcinogenesis [37,38,39]. It was reported that chemokines were implicated in many aspects of carcinogenesis like tumor growth, angiogenesis, and metastatic development [38]. Some tumor cells utilized factors to promote tumor development and progression, besides controlling the expression of chemokines to aid in the recruitment of inflammatory cells [40].

Inflammation promotes tumors and intrinsic and extrinsic factors generating chronic inflammation leading to carcinogenesis/tumorigenesis [41,42]. Previous studies from our laboratory analyzed those genes related to the nervous system [43], growth factor [7], and adhesion molecules [44] that were changed due to the effect of ionizing radiation and estrogen. This study aimed to analyze the expression of genes associated with inflammation such as the interleukin 1 receptor type 1 (*IL1R1*), the interleukin 1 receptor antagonist *(IL1RN*), the Interleukin 1 Receptor Accessory Protein (*ILRAP*), the interleukin 6 cytokine family signal transducer (*IL6ST*), the C-X-C motif chemokine ligand 3 (*CXCL3*), the C-X-C motif chemokine ligand 5 (*CXCL5*), and the C-X-C motif chemokine ligand 6 (*CXCL6*) in a previously developed estrogen and radiation experimental breast cancer model named after the Alpha Model [45]. Moreover, such genes were compared with clinical parameters of datasets available online. Analyzing these genes associated with inflammation will help to clarify the role of these mediators in breast carcinogenesis induced by estrogen or radiation.

## 2. Results

Genes associated with inflammation, including *IL1R1*, *IL1RN*, *ILRAP*, *IL6ST*, *CXCL3*, *CXCL5*, and *CXCL6*, were chosen based on microarray data obtained from the genetic material of Alpha model cells. The gene expression in the Alpha model was contrasted with online databases for breast cancer patients. Analysis was performed on the expression of these genes in the innate immune system cells, in subtypes of breast cancer patients, and concerning the estrogen receptor alpha. It also assessed the expression of these inflammatory genes in breast cancer patient prognosis.

### 2.1. Differential Gene Expression in a Radiation and Estrogen Experimental Breast Cancer Model

**Figure 1:**
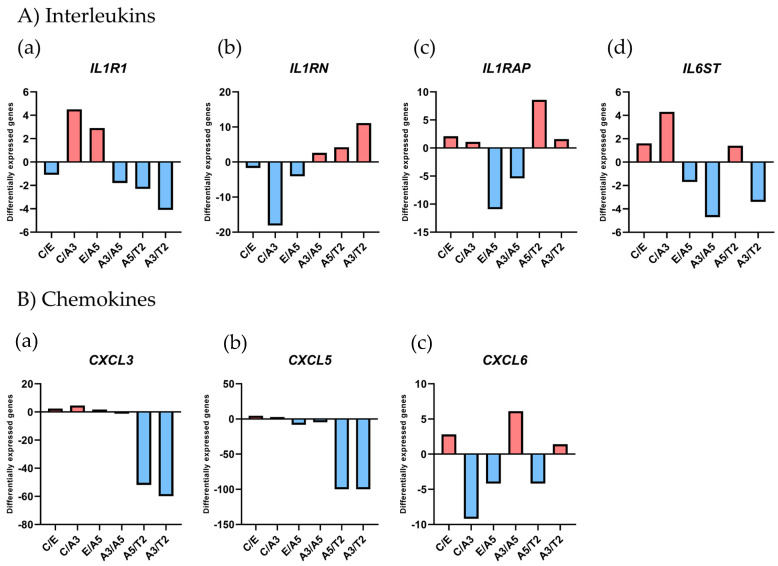
Relative gene expression from the Affymetrix array (U133A) in radiation- and estrogen-induced experimental breast cancer model. The analyzed genes were (**A**) the interleukins such as (**a**) the Interleukin 1 Receptor Type 1 (*IL1R1*), (**b**) the Interleukin 1 Receptor Antagonist (*IL1RN*), (**c**) Interleukin 1 Receptor Accessory Protein (*ILRAP*), and (**d**) Interleukin 6 Cytokine Family Signal Transducer (*IL6ST*), (**B**) chemokines such as (**a**) the C-X-C motif chemokine ligand 3 (*CXCL3*), (**b**) C-X-C motif chemokine ligand 5 (*CXCL5*), and (**c**) C-X-C motif chemokine ligand 6 (*CXCL6*) in the following cell lines: MCF-10F/Estrogen (Ct/E); Control/Alpha3 (Ct/A3); Estrogen/Alpha5 (E/A5); Alpha3/Alpha5 (A3/A5); Alpha5/Tumor2 (A5/T2); and Alpha3/Tumor2 (A3/T2). The graphs were obtained from a Cluster-dendrogram repository of gene expression from our laboratory for this article.

The differential gene expression associated with the inflammatory response by using the Affymetrix array U133A was analyzed in the following design: the MCF-10F/Estrogen (Ct/E), Control/Alpha3 (Ct/A3), Estrogen/Alpha5 (E/A5), Alpha3/Alpha5 (A3/A5), Alpha5/Tumor2 (A5/T2), and Alpha3/Tumor2 (A3/T2), as seen in Figure 1 and Table 1.

Results in Figure 1 related to interleukin gene expression show that *IL1R1* gene expression was higher in the irradiated A3 cell line than in the control (Ct) and tumorigenic (T2) cell lines. Furthermore, A5 cells (with radiation and estrogen) had higher *IL1R1* gene expression than in the estrogen (E) cell line. In Figure 1(Ab)), it is shown that the *IL1RN* gene expression was higher in the Ct and T2 cells than in the A3 cell line. On the other hand, the *IL1RAP* gene expression was higher in the E, A3, and T2 than the A5 cell lines (Figure 1(Ab). Finally in Figure 1(Ad)), the signal transducer *IL6ST* was shown to have a higher gene expression in the A3 compared to other cell lines.

Results from Figure 1B indicated that chemokines *CXCL3* (a) and *CXCL5* (b) gene expression was higher in the A3 and A5 than the T2 cell lines, whereas *CXCL6* (c) gene expression was higher in the Ct and A5 than the A3 cell lines.

### 2.2. Gene Expression Levels in Tumor Versus Normal Tissues across Breast Cancer Subtypes

**Figure 2:**
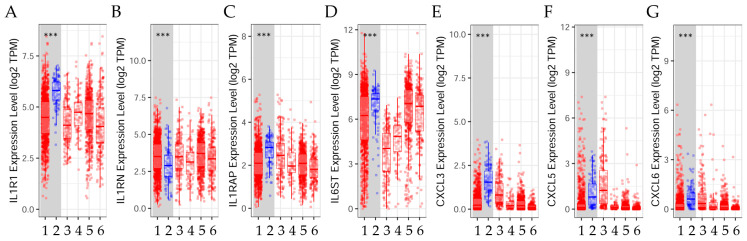
Gene expression levels in breast tumor (red) and normal (blue) tissues across breast cancer subtypes. The box plots show the distribution of gene expression levels of (**A**) the Interleukin 1 Receptor Type 1 (*IL1R1*), (**B**) the Interleukin 1 Receptor Antagonist (*IL1RN*), (**C**) the Interleukin 1 Receptor Accessory Protein (*ILRAP*), (**D**) the Interleukin 6 Cytokine Family Signal Transducer (*IL6ST*), (**E**) the C-X-C motif chemokine ligand 3 (*CXCL3*), (**F**) the C-X-C motif chemokine ligand 5 (*CXCL5*), and (**G**) the C-X-C motif chemokine ligand 6 (*CXCL6*) in tumors versus normal tissues (Wilcoxon test, ***: *p* < 0.001) estimated by TIMER2.0 (http://timer.cistrome.org) in breast invasive carcinoma [46], accessed on 14 April 2024. 1: Tumor (*n* = 1093), 2: Normal (*n* = 112), 3: Basal. Tumor (*n* = 190), 4: Her2. Tumor (*n*= 82), 5: Luminal A. Tumor (*n* = 564), and 6: Luminal B. Tumor (*n* = 217).

The results in Figure 1 showed that there is variation in the expression of inflammatory genes due to the different stages of transformation of the breast cells in the Alpha model. To determine the expression of inflammatory genes in samples of healthy people and those with breast cancer, the TIMER2.0 online patient database platform was used. Results in Figure 2 indicated that *IL1RN* expression was higher in the tumor compared to the adjacent normal tissue. However, *IL1R1*, *ILRAP*, *IL6ST*, *CXCL3*, *CXCL5*, and *CXCL6* gene expression was higher in normal adjacent tissue than in breast tumor tissue. The statistical significance was calculated using the Wilcoxon test, *p* < 0.001.

### 2.3. Gene Expression Associated with Inflammation and ESR1 Gene Expression in Breast Cancer Subtypes

**Figure 3:**
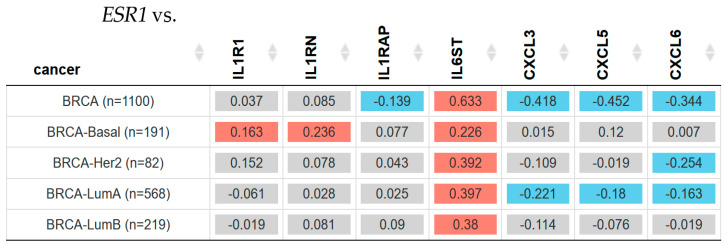
Correlation between the estrogen receptor alpha gene (*ESR1*) and genes related to inflammation such as the Interleukin 1 Receptor Type 1 (*IL1R1*), the Interleukin 1 Receptor Antagonist (*IL1RN*), the Interleukin 1 Receptor Accessory Protein (*ILRAP*), the Interleukin 6 Cytokine Family Signal Transducer (*IL6ST*), the C-X-C motif chemokine ligand 3 (*CXCL3*), the C-X-C motif chemokine ligand 5 (*CXCL5*), and the C-X-C motif chemokine ligand 6 (*CXCL6*) in breast invasive carcinoma subtypes. The heatmap table gives the purity-adjusted partial Spearman’s rho value as the degree of their correlation. The red color indicates a statistically significant positive correlation (Spearman’s, *p* < 0.05), blue indicates a statistically significant negative correlation (Spearman’s, *p* < 0.05), and gray denotes a non-significant result. Estimated by TIMER2.0 (http://timer.cistrome.org) in breast cancer patients [46], accessed on 14 April 2024.

The hormonal estrogen receptor (ER) is the most important biomarker in breast oncology. Consequently, the association between the selected inflammatory genes and the gene that encodes ERα (*ESR1*) was assessed using the same patient database from the TIMER2.0 platform. Correlation between *ESR1* and *IL1R1*, *IL1RN*, *ILRAP*, *IL6ST*, *CXCL3*, *CXCL5*, and *CXCL6* in breast invasive carcinoma can be seen in Figure 3.

Figure 3 shows there was a significant (Spearman test, *p* < 0.05) positive correlation between *IL1R1*, *IL1RN*, and *ESR1* gene expression in Basal patients; however, there was no significant difference in *IL1RAP* correlation in any subtype of breast cancer patients, but all were positive when the correlation was made between *IL6ST* and *ESR1* gene expression in all breast cancer subtypes such as Basal, Her2, Luminal A, and Luminal B.

On the other hand, the results showed that there was a statistically negative significance (Spearman test, *p* < 0.05) between *ESR1* and *CXCL3* and *CXCL5* gene expression in Luminal A patients. Additionally, *CXCL6* gene expression and *ESR1* showed a negative significant (*p* < 0.05) correlation in breast cancer Her2 and Luminal A patients.

### 2.4. Gene Expression and ER Status in TCGA Breast Cancer

**Figure 4:**
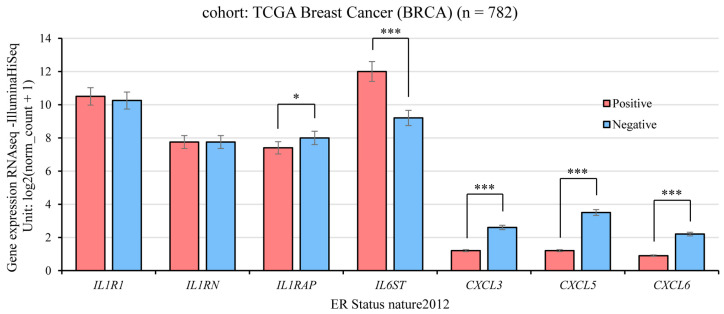
Gene Expression and Estrogen Receptor Status in TCGA Breast Cancer. The graphs show the gene expression of the Interleukin 1 Receptor Type 1 (*IL1R1*), the Interleukin 1 Receptor Antagonist (*IL1RN*), the Interleukin 1 Receptor Accessory Protein (*ILRAP*), the Interleukin 6 Cytokine Family Signal Transducer (*IL6ST*), the C-X-C motif chemokine ligand 3 (*CXCL3*), the C-X-C motif chemokine ligand 5 (*CXCL5*), and the C-X-C motif chemokine ligand 6 (*CXCL6*) in breast invasive carcinoma, phenotypically classified by nature 2012 for estrogen receptor status (one-way ANOVA, *: *p* < 0.05 and ***: *p* < 0.001). The red color indicates a positive estrogen receptor status, and the blue color indicates a negative estrogen receptor status. Raw data were extracted from the University of California, Santa Cruz, UCSC Xena Functional Genomics Explorer (http://xena.ucsc.edu/) [47], accessed on 14 April 2024.

UCSC Xena [47] web is a tool that allows correlation between genomic and/or phenotypic data. We evaluate the expression of inflammation genes (genomic data) with the ER status (phenotypic data) in breast cancer patients as seen in Figure 4.

Results indicated that *IL1R1* and *IL1RN* gene expression showed no significant difference between ER-positive or ER-negative breast cancer patients (One-way ANOVA *p* = 0.9597 and *p* = 0.4699, respectively). But *IL1RAP* (*p* = 4.234 × 10^−11^), *CXCL3* (*p* = 0), *CXCL5* (*p* = 0), and *CXCL6* (*p* = 0) gene expression levels had higher expression in ER-negative than ER-positive ones; whereas *IL6ST* was the only gene whose expression was higher in ER-positive cancer patients (*p* = 0).

### 2.5. Gene Expression and Clinical Survival in Breast Cancer Subtypes

**Figure 5:**
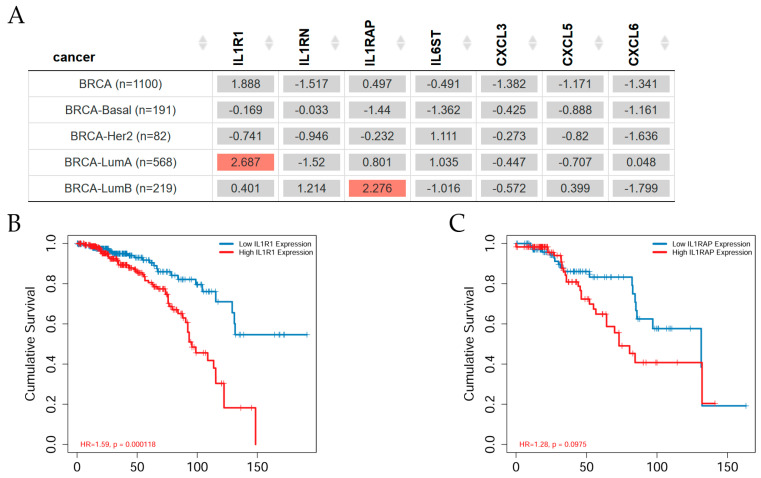
Survival and breast invasive carcinoma subtypes. (**A**) The heatmap table shows the normalized coefficient of the genes in the Cox model; genes such as the Interleukin 1 Receptor Type 1 (*IL1R1*), the Interleukin 1 Receptor Antagonist (*IL1RN*), the Interleukin 1 Receptor Accessory Protein (*ILRAP*), the Interleukin 6 Cytokine Family Signal Transducer (*IL6ST*), the C-X-C motif chemokine ligand 3 (*CXCL3*), the C-X-C motif chemokine ligand 5 (*CXCL5*), and the C-X-C motif chemokine ligand 6 (*CXCL6*) in breast cancer patients. The red color indicates a statistically significant increased risk (Z-score, *p* < 0.05) and gray denotes a non-significant result. The Kaplan–Meier (KM) curve shows cumulative survival versus time to follow-up of (**B**) *IL1R1* and (**C**) *IL1RAP* gene expression levels. Survival probability is represented on the y-axis, and time (in months) on the x-axis. The blue curve corresponds to low gene expression and the red curve to high gene expression. Survival estimated by TIMER2.0 (http://timer.cistrome.org) in breast invasive carcinoma patients [46], accessed on 14 April 2024.

The Gene Outcome module of the TIMER2.0 web dataset was used to evaluate whether the expression level of the genes in this study could have any predictive value for breast cancer overall survival (Figure 5).

Figure 5A shows that *IL1R1* gene expression presented a significant (Z-score, *p* < 0.05) increased risk in breast invasive carcinoma Luminal A patients, whereas *IL1RAP* gene expression depicted an increased risk in Luminal B patients. However, *IL1RN*, *IL6ST*, *CXCL3*, *CXCL5*, and *CXCL6* gene expression levels were non-significant concerning clinical survival of breast cancer patients according to the Cox proportional Hazard model. Figure 5B shows Kaplan–Meier (KM) survival curves as indicating a better survival for low *IL1R1* expression in breast invasive carcinoma Luminal A patients, whereas high *IL1R1* expression showed no survival at 150 months. However, high *IL1RAP* expression showed a decreased survival in comparison with low *IL1RAP* expression in Luminal B patients as seen in Figure 5C. This difference occurred between 30 and 130 months in the KM Curve analysis. After this time, Luminal B patients with high and low *IL1RAP* expression showed a value of 0.2 on the cumulative survival axis. After 130 months, Luminal B patients with high or low *IL1RAP* had a 20% probability of surviving.

### 2.6. Gene Expression and Immune Infiltration in Breast Cancer

**Figure 6:**
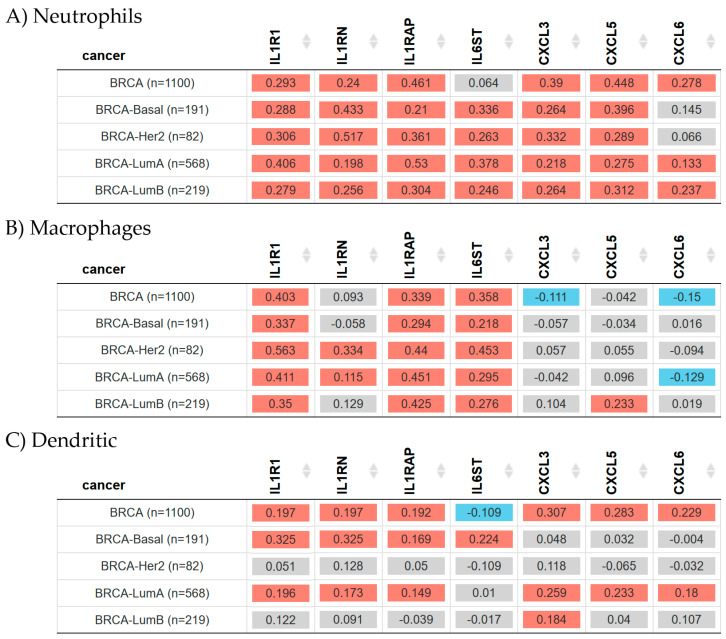
Cell infiltrates and the expression of genes associated with inflammation across different breast invasive carcinoma subtypes. The heatmap table shows the correlation between (**A**) neutrophil, (**B**) macrophage, (**C**) dendritic cell infiltration levels and the Interleukin 1 Receptor Type 1 (*IL1R1*), the Interleukin 1 Receptor Antagonist (*IL1RN*), the Interleukin 1 Receptor Accessory Protein (*ILRAP*), the Interleukin 6 Cytokine Family Signal Transducer (*IL6ST*), the C-X-C motif chemokine ligand 3 (*CXCL3*), the C-X-C motif chemokine ligand 5 (*CXCL5*), and the C-X-C motif chemokine ligand 6 (*CXCL6*) gene expression levels in breast invasive carcinoma subtypes. The red color indicates a statistically significant positive correlation (Spearman’s, *p* < 0.05), the blue color indicates a statistically significant negative correlation (Spearman’s, *p* < 0.05), and gray denotes a nonsignificant result. Raw data were extracted from TIMER2.0 (http://timer.cistrome.org) in breast cancer patients [46], accessed on 14 April 2024.

A tumor-infiltration analysis was performed to estimate the association between the three innate immunity cell types such as neutrophils, macrophages, and dendritic cells, and the genes in this study in breast cancer subtypes as seen in Figure 6. For this purpose, the “Gene” module of the “Immune” component of the TIMER2.0 web page was used.

Results indicated there was a statistically significant (Spearman’s, *p* < 0.05) positive correlation between neutrophils and *IL1R1*, *IL1RN*, *IL1RAP*, *IL6ST*, *CXCL3*, and *CXCL5* gene expression in Basal, Her2, Luminal A, and Luminal B patients (Figure 6A). Additionally, *CXCL6* gene expression showed a positive correlation with breast invasive carcinoma Luminal A and Luminal B patients.

Macrophage infiltration (Figure 6B) indicated a statistically significant (Spearman’s, *p* < 0.05) positive correlation with *IL1R1*, *IL1RAP*, and *IL6ST* gene expression in Basal, Her2, Luminal A, and Luminal B patients. *IL1RN* was positively correlated with macrophage infiltration in breast cancer Her2 and Luminal A patients. *CXCL5* gene expression was positively correlated with such infiltration in Luminal B patients. However, *CXCL6* was negatively correlated with this infiltration in the Luminal A subtype.

Dendritic cells showed a significant (Spearman’s, *p* < 0.05) positive correlation with *IL1R1*, *ILRN*, and *ILRAP* in breast invasive carcinoma-Basal and Luminal A patients (Figure 6C). *IL6ST* gene expression was positively correlated with such infiltration in breast invasive carcinoma-Basal with a similar significance. *CXCL3*, *CXCL5*, and *CXCL6* gene expression levels were positively correlated with dendritic cell infiltration in Luminal A patients; additionally, *CXCL3* expression was also positively correlated with dendritic cells in Luminal B patients.

**Figure 7:**
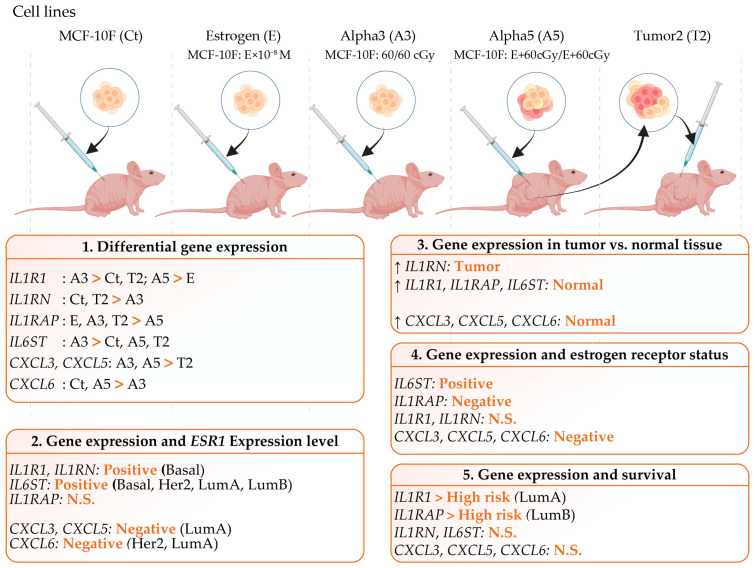
Gene signature associated with inflammation in an experimental breast cancer model induced by ionizing radiation and estrogen. Genes analyzed in this figure: *IL1R1*, *IL1RAP*, *IL1RN*, *IL6ST*, *CXCL3*, *CXCL5*, and *CXCL6* in non-tumorigenic cell lines such the MCF-10F (Ct), the estrogen (E), the Alpha3 (A3), and tumorigenic cell lines such as the Alpha5 (A5), and the Tumor2 (T2), which were injected in the nude mice. Analysis of (1) Differential gene expression of genes in the E, the A3, the A5, and the T2 breast cancer cell lines. (2) Correlation between the estrogen receptor alpha gene (*ESR1*) and the list of genes under study in breast invasive carcinoma patients. (3) Gene expression in tumor versus normal tissue. (4) Gene expression and estrogen receptor status in breast cancer patients. (5) Gene expression and patients’ survival.

Gene signatures associated with inflammation in an experimental breast cancer model induced by ionizing radiation and estrogen can be seen in Figure 7. Genes analyzed in this study were *IL1R1*, *IL1RAP*, *IL1RN*, *IL6ST*, *CXCL3*, *CXCL5*, and *CXCL6* in non-tumorigenic cell lines such the MCF-10F (Ct), the estrogen (E), the Alpha3 (A3), and tumorigenic cell lines such as the Alpha5 (A5), and the Tumor2 (T2). This work also considered the analysis of (1) Differential gene expression of genes in the E, the A3, the A5, and the T2 breast cancer cell lines. (2) Correlation between the estrogen receptor alpha gene (*ESR1*) and the list of genes under study in breast invasive carcinoma patients. (3) Gene expression in tumor versus normal tissue. (4) Gene expression and estrogen receptor status in breast cancer patients. (5) Gene expression and patients’ survival.

## 3. Discussion

The results related to interleukins indicated that *IL1R1* gene expression was higher in the A3 and A5 than in other cell lines indicating that such gene expression increased in response to radiation in the early stages of cell transformation and influenced tumor formation in this experimental model. It was reported that *IL1R1* could be expressed in numerous cell types by reacting to both IL-1α and IL-1β. Even though these two cytokines comparably transduce signaling, the role of IL-1α has been debated [48,49,50,51,52,53], but IL-1β was shown to promote tumor growth in cancer [54,55]. Few antecedents connect IL-1R1 signaling with its IL-1α/β ligands in response to radiation. However, it has been observed that in a murine lung model, the gamma irradiation with a single (20 Gy) or fractionated doses (4 Gy/day) increased IL-1β within 1 h [56].

The *IL1RN* is the antagonist of *IL1R1* whose gene expression was higher in the Ct and the T2 cell than in the A3 cell line. *IL1RN* has been described as an inhibitor of inflammation [57], and *IL1RN* gene expression has been reported in advanced stages of cancer that could be related to an evasion of the immune response, a mechanism that some cancers use to continue their progression. The *IL1RN* production has also been reported to increase in response to low doses of either UV or ionizing irradiation in cultured human orbital fibroblasts derived from patients with active Graves’ ophthalmopathy [58].

On the other hand, *IL1RAP* gene expression increases in the E, A3, and T2 cell lines. Probably resulting from exposure to estrogens and radiation or the tumor microenvironment developed in the animal. Similarly, a study showed that *IL1RAP* was upregulated in normal human fibroblast cultures carbon ion-irradiated or in response to ionizing radiation in the liver indicated after 2 Gy proton exposure [59].

The *IL6ST* gene expression was higher in A3 than in the Ct, A5, and T2 cell lines, indicating the importance of the ionizing radiation effect during the first stages of the carcinogenic process. The role of several interleukins in inflammation has been highly significant in the study of various cancers especially *IL6ST,* which is upregulated through its development [60,61]. Results indicated that chemokines *CXCL3* and *CXCL5* gene expression were higher in the A3 and A5 than in T2 cell lines, indicating that they were important for cancer initiation either by the effect of radiation or estrogen. The response of chemokines to ionizing radiation is supported by a study that showed that CXCL3 and CXCL5 gene expression was upregulated in the skin and dorsal root ganglia after ultraviolet-B exposure [62].

Concerning the clinical studies, gene expression was analyzed in normal and tumor tissues across breast cancer subtypes and results indicated that *IL1RN* expression was higher in the tumor compared to the adjacent normal tissue. According to other authors [57], *IL1RN* was released upon cell death, limiting the pro-inflammatory action of tissue damage. Thus, high *IL1RN* expression could have either good [18,21,24,25,26,27] or bad cancer prognosis [26,28,29]. However, *IL1R1*, *ILRAP*, *IL6ST*, *CXCL3*, *CXCL5*, and *CXCL6* gene expression levels were higher in normal adjacent tissue than in breast tumor tissue indicating that the surrounding non-tumor tissue could also present an inflammation. Thus, potentially invasive and metastatic tumors may gain an advantage in recruiting tumor-promoting inflammatory cells that bring growth-stimulating factors to the surviving cells [63].

The association between *ESR1* and the genes in the study was examined since the ER is crucial to the biology of breast cancer. In this sense, there was a positive correlation between *IL1R1* or *IL1RN* and *ESR1* gene expression in Basal subtypes; however, *IL1RAP* showed no significant difference in any breast cancer subtype. When the correlation was made between *ESR1* and *IL6ST* gene expression, they were positive in all breast invasive carcinoma subtypes, i.e., positive for Basal, Her2, Luminal A, and Luminal B breast cancer patients. These results provided a good marker of inflammations due to the high values obtained through the correlation between *ESR1* and *IL6ST*.

The relationship between *ESR1* gene expression with *IL1R1* or *IL1RN* genes has not been described for breast cancer. However, according to the results obtained in a murine model of psoriasis, it is inferred that the expression of estrogen receptors and levels of inflammation are directly related. Mice without endogenous ovarian hormones exhibited exacerbated psoriatic inflammation due to increased production of IL-17A and IL-1β (IL1R1 ligand), which was reversed by exogenously added estradiol [64].

The positive correlation between *IL6ST* and *ESR1* in all breast cancer subtypes might be due to IL6ST interaction with the estrogen signaling pathway in ER-positive breast cancer as described by Mosly et al. [65]. Such a study also indicated that cytokines could inhibit and stimulate cell proliferation via IL6ST in breast cancer cells. The high IL6ST expression was associated with cell inhibition and low expression with stimulation in ER-positive breast cancer. Such a mechanism has not been described yet.

However, in patients with Luminal A breast invasive carcinoma, there was a negative correlation found between *ESR1* and *CXCL3*, *CXCL5*, and *CXCL6* gene expression; and in Her-2 patients, the same relationship was seen between *ESR1* and *CXCL6*. These findings are in concordance with the ER status analysis and show an inversely proportional link between the expression of these chemokines and the expression of *ESR1.*

Gene expression associated with ER status in breast cancer patients was analyzed and results indicated that *IL1R1* and *IL1RN* gene expression levels were non-significant among patients, whereas those with high *IL6ST* expression levels had a positive ER status. However, patients with high *IL1RAP*, *CXCL3*, *CXCL5*, and *CXCL6* gene expression had a negative ER status.

It is unclear how differently physiologically high or low levels of estrogens modify ER activity to impact immune response gene expression. They might probably create different ER-containing transcriptional complexes, which produce distinct patterns of epigenetic marks on genes within functional pathways that either stimulate or inhibit inflammation [66]. For example, the nuclear factor NF-kappaB pathway is considered a prototypical proinflammatory signaling pathway, largely based on the role of NF-kappaB in the expression of proinflammatory genes including cytokines, chemokines, and adhesion molecules [66]. ERα participates in NF-κB transcriptional complexes, often with inhibitory effects on both ER and NF-κB-mediated transcriptional activity [67]. The inhibition of the NF-κB pathway by ERs often limits the extent of the inflammatory response and occurs by several mechanisms [68]. On the other hand, ERs also have been reported to have regulatory interactions with many other transcription factors (e.g., SP1, AP-1) involved in innate immune pathways. However, the mechanism by which the expression of *ESR1* or ERα affects the immune-specific response of the genes studied here has not been elucidated.

The expression level of the genes analyzed in this study may have a predictive value for breast cancer overall survival since it indicated that *IL1R1* and *ILRAP* gene expression presented an increased risk in breast invasive carcinoma-LumA and -LumB patients. However, *IL1RN*, *IL6ST*, *CXCL3*, *CXCL5*, and *CXCL6* gene expression levels were non-significant concerning clinical survival of breast cancer patients. A better survival was found for low *IL1R1* expression LumA patients than in high IL1R1 expression patients, who did not show survival at 150 months. LumB patients with high *IL1RAP* expression had a lower survival rate than those with low *IL1RAP* expression. The difference in patient survival was observed between 30 and 130 months and, after this time, the patients with high and low *IL1RAP* expression showed the same value of 0.2 on the cumulative survival axis. In summary, the analysis of breast cancer overall survival indicated that a high level of *IL1R1* expression in LumA patients presented no survival after 150 months. Patients with high *IL1RAP* expression had their survival rate reduced by 80% within 150 months.

Although our results indicate that the inflammatory genes chosen in this paper are expressed in breast cancer tissue, the role of other components of the tumor microenvironment remains elusive. According to Affymetrix array results, the breast cancer cells by themselves could express genes related to inflammation in response to radiation alone or combined with estrogens. To understand the role of the chosen genes in the innate immune response, we considered the correlation between the expression of genes associated with inflammation and neutrophils, macrophages, and dendritic cells in breast cancer subtypes.

Neutrophil infiltration had a direct relationship with the expression of *IL1R1*, *IL1RN*, *IL1RAP*, *IL6ST*, *CXCL3*, *CXCL5*, and *CXCL6* in most of the subtypes of the breast cancer patients analyzed. Such results followed the literature that indicated that interleukins and chemokines and their receptor families played a crucial role in facilitating the migration of leukocytes to sites of inflammation [69]. Prolonged inflammation creates an ideal environment for the development and proliferation of tumor cells. Chemokines impact various aspects of tumor behavior, including transformation, survival, growth, invasion, and metastasis by regulating interactions between tumor cells, angiogenesis, and leukocytes [70,71].

On the other hand, the macrophage infiltration showed a positive correlation with *IL1R1*, *IL1RAP*, and *IL6ST* gene expression in all subtypes of patients with breast cancer. Particularly, *IL1RN* was positively correlated with this infiltration in Her2 and LumA patients. *CXCL5* gene expression was positively correlated only in LumB patients, whereas *CXCL6* was negatively correlated with macrophage infiltration in the Luminal A subtype.

Dendritic cell infiltration showed a positive correlation with *IL1R1*, *ILRN*, and *ILRAP* gene expression in breast cancer Basal and Luminal A patients. *IL6ST* gene expression positively correlated with such infiltration in breast cancer Basal patients. Thus, there is a connection between the presence of dendritic cells and the interleukin family gene expression within the tumor. For the chemokine family genes, *CXCL3*, *CXCL5*, and *CXCL6,* gene expression was positively correlated with dendritic cell infiltration in breast invasive carcinoma-Luminal A patients. Additionally, *CXCL3* expression positively correlated with dendritic cells in Luminal B patients. These findings suggest that, in contrast to neutrophilic infiltration, which was more transversal, the infiltration of both macrophages and dendritic cells mainly depends on the subtype of breast cancer.

The expression of ERα, progesterone receptor (PR), and human epidermal growth factor receptor 2 (HER2) influenced the number and variety of immune cells [72]. A study [73] revealed there were five heterogeneous immune subtypes among ER+/PR–/HER2− breast cancer and that they had important implications for clinical translations. ER activity was shown to increase or decrease innate immune signaling pathways in dendritic cells and macrophages. Thus, estradiol and ERs were reported to exert either positive or negative regulatory effects on pro-inflammatory cytokine production that varied with the cell type or estrogen dose [66].

A study [66] indicated that both estradiol and ER signaling regulated the inflammatory pathways in innate immune cells, such as dendritic cells and macrophages. Sometimes, ER signaling mitigated those pathways, even in environments where estrogen levels were low. Higher physiological or supraphysiological levels of estrogens mostly fostered anti-inflammatory responses, which helped to diminish inflammation. Similarly, ER signaling either promoted or hindered the growth of new immune cells through its impact on hematopoietic progenitors [66]. Additionally, another study in a murine model of psoriasis suggests that the expression of estrogen receptors and levels of inflammation are directly related. Estradiol suppressed IL-1β production from neutrophils and macrophages in mice both in vivo and in vitro and from human neutrophils in vitro [64]

Factors such as radiation and hormones can damage tissues, but repairing damaged tissue generates an inflammatory response that causes quantitative and qualitative changes in the immune cell population at the site of injured tissue. When inflammation becomes chronic, it can lead to cell mutation and proliferation, often creating a favorable microenvironment for cancer development [14].

Chemokines and interleukin molecules are part of that microenvironment and have been implicated in many aspects of carcinogenesis. Results of this study show that both the interleukin 1 gene family and chemokines are important for the development and progression of breast cancer. These genes can be expressed in normal and tumor breast tissue and generate immune infiltration cells in the tumor microenvironment. These responses depend on the breast cancer subtype (therefore of the molecular markers) and some genes are characterized by their high expression levels and the relation to poor survival.

A limitation of this work is that the analysis based on bioinformatics tools includes a population of patients whose origin of cancer is not determined. Therefore, the influence of ionizing radiation and estrogen could be a possibility of transformation. It is important to note that this work contributes to emphasizing the importance of genes associated with inflammation in the process of initiation and progression of breast cancer. The findings in this research may pave the way for novel therapeutic approaches targeting BC.

## 4. Materials and Methods

**Figure 8:**
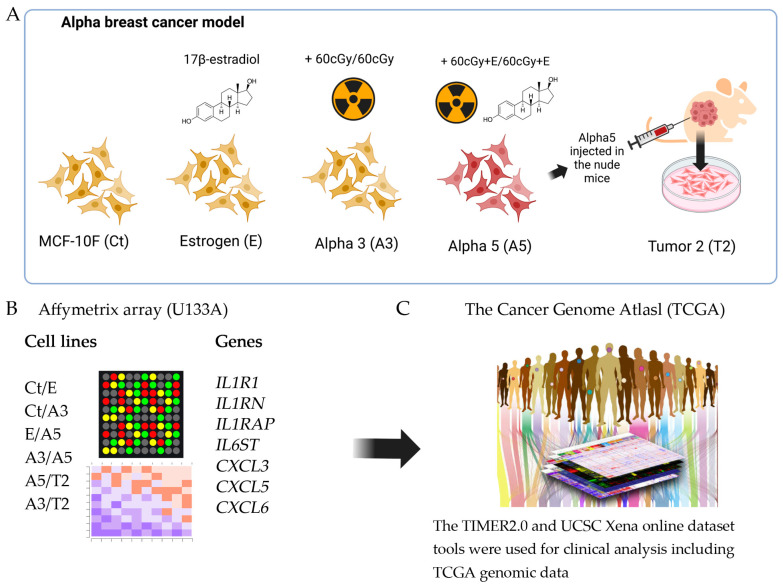
Schematic representation of the methodology used in this work: (**A**): the parental cell line MCF-10F (Ct); estrogen cell line (E), MCF-l0F continuously grown with 17β-estradiol, a malignant and non-tumorigenic cell line (60/60 cGy) named Alpha3 (A3), a tumorigenic cell line (60cGy + E/60cGy + E) named Alpha5 (A5); and the Tumor2 cell line (T2), which was developed by injecting an Alpha5 cell line xenograft into nude mice. (**B**) groups compared on the Affymetrix (U133A) oligonucleotide microarray to evaluate the differentially expressed genes. (**C**) The TIMER2.0 and UCSC Xena platforms that use the Databases of The Cancer Genome Atlas (TCGA) available online were chosen to analyze the inflammatory genes of this work.

Figure 8 shows a simplified scheme of the methodology of this work. Briefly, Figure 8A shows the alpha model of breast cancer: the cells used and the conditions to transform them developed by Calaf and Hei [45]. Figure 8B shows the groups compared on the Affymetrix (U133A) oligonucleotide microarray to evaluate the differentially expressed genes. Finally, from the results of the Affymetrix analysis, the inflammatory genes were chosen and then compared with the databases of The Cancer Genome Atlas (TCGA) using the platforms available online (Figure 8C).

Figure 8 represents the methodology that characterizes the estrogen and radiation model. The research scheme was presented in the following order (1) Differential gene expression in a radiation and estrogen experimental breast cancer model. (2) Gene expression associated with inflammation and estrogen receptor alpha gene (*ESR1*) in breast cancer subtypes. (3) Gene expression and ER status in TCGA breast cancer. (4) Gene expression levels in tumor versus normal tissues across breast cancer subtypes. (5) Gene expression and clinical survival in breast cancer subtypes. (6) Gene expression and immune infiltration in breast cancer.

### 4.1. An Experimental Radiation- and Estrogen-Induced Breast Cancer Model, Named Alpha Model

The human breast epithelial cell line MCF-10F (ATCC), known for its immortal characteristics and developed by Soule et al. [74], was exposed to low doses of high linear energy transfer (LET) radiation (150 keV/m) in an environment with or without 17ß-estradiol using the 4 Me V van de Graaff accelerator at the Columbia University Radiological Research Facilities, as previously mentioned [45]. When the MCF-10F cell line was exposed to either a single 60 cGy dose or two 60 cGy doses of alpha particles, different stages of transformation were observed, including changes in morphology, elevated cell proliferation in comparison with the control, anchorage-independent growth, and the ability to invade before becoming tumorigenic in nude mice [45].

### 4.2. Cell Lines

The MCF-10F cell line was cultured in DMEM/F-12 (1:1) media enhanced with antibiotics such as 100 U/mL penicillin, 100 g/mL streptomycin, and 2.5 g/mL amphotericin B, all from Life Technologies, Grand Island, NY, USA. Additionally, the media were enriched with 0.02 g/mL of epidermal growth factor from Collaborative Research, Bedford, MA, USA, 0.5 g/mL of hydrocortisone (from Sigma, St. Louis, MO, USA), and 10 g/mL along with 5% equine serum from Biofluids, Rockville, MD, USA [45,74,75,76,77]. The cell lines used in the present study from the Alpha Model were (i) the parental cell line MCF-10F (Control); (ii) an estrogen cell line (E), MCF-l0F continuously grown with 17β-estradiol (Sigma Chemical Co., St. Louis, MO, USA) at 10^−8^ M; (iii) a malignant and non-tumorigenic cell line (60/60 cGy) named Alpha3 (A3); (iv) a tumorigenic cell line (60cGy + E/60cGy + E) named Alpha5 (A5); and (v) the Tumor2 cell line (T2), which was developed by injecting an Alpha5 cell line xenograft into nude mice. Phenotypical studies of an experimental breast cancer model: Alpha Model can be seen in Table 2. These cell lines were cultured for up to 10 months and the scheme of animals injected with the cell lines indicated that E and A3 did not form mammary tumors, whereas A5 and T2 were tumorigenic in the model and SCID animal [44].

### 4.3. Affymetrix (U133A) Oligonucleotide Microarray

Using an Affymetrix (U133A) oligonucleotide microarray (Affymetrix, Santa Clara, CA, USA), differentially expressed genes were measured to identify their expression associated with the inflammatory response. The Gene expression from the array was quantitatively assessed using the Affymetrix GeneChip Operating Software (GCOS) version 1.0 ST and the analysis was performed using the SPLASH (structural pattern localization analysis by sequential histograms) discovery approach with a false discovery rate of 0.05 [81]. The experiment of the Affymetrix U133A oligonucleotide microarray was carried out once and contained 14,500 genes. Profiling of differentially expressed genes was evaluated in the following cell lines: MCF-10F/Estrogen (Ct/E), Control/Alpha3 (Ct/A3), Estrogen/Alpha5 (E/A5), Alpha3/Alpha5 (A3/A5), Alpha5/Tumor2 (A5/T2), and Alpha3/Tumor2 (A3/T2).

### 4.4. Bioinformatics

Bioinformatics tools explain the genes under study with a variety of clinical parameters. For such a purpose, the Tumor Immune Estimation Resource v2.0 (TIMER2.0, http://timer.cistrome.org/, accessed on 14 April 2024) [46] was used. It is an online dataset with three main components (The Immune, the Exploration, and the Estimation components), each having different modules. The Gene Corr module of the Cancer Exploration component estimated the correlation of genes in various breast invasive carcinoma subtypes such as Basal, Her2, Luminal A (LumA), and Luminal B (LumB). The Gene DE module of the Exploration component was used to estimate the differential gene expression between tumors and adjacent normal tissues. Survival was provided by the Gene Outcome module of the Cancer Exploration component. The Gene module of the Immune Association component presented an estimate of immune infiltration levels.

The University of California, Santa Cruz, through the UCSC Xena Functional Genomics Explorer (http://xena.ucsc.edu/, accessed on 14 April 2024) [47], another online tool, provided functional genomic datasets for correlations between genomic and/or phenotypic data such as the ER status.

Both tools TIMER2.0 and UCSC Xena datasets include data from The Cancer Genome Atlas (TCGA) where the abbreviation BRCA stands for TCGA Breast Cancer (breast invasive carcinoma).

### 4.5. Statistical Analysis

The purity-adjusted Spearman’s rho test was used for the correlation of genes, the Wilcoxon test was employed for the differential gene expression between tumors and adjacent normal tissue analysis, the Spearman’s test was used for immune infiltration levels, and the log-rank test was used for Kaplan–Meier survival data analysis, whereas the Cox proportional hazard model was used to evaluate the outcome significance of gene expression (Z-score test); these tests were estimated by TIMER2.0, reference number (54), whereas UCSC Xena employed One-way ANOVA test for the estrogen receptor status analysis, reference number (55). Numerical data are expressed as the means ± standard error of the mean (SEM). Comparisons between 2 groups were made using the ANOVA with Dunnett’s test between several treatment groups and the controls. A value of *p* < 0.05 was considered to indicate a statistically significant difference.

## 5. Conclusions

These findings suggest a gene signature associated with ionizing radiation and estrogen that suggests important genetic changes in breast epithelial cells. According to the results obtained by the model, *IL1R1*, *IL6ST*, *CXCL3*, *CXCL5*, and *CXCL6* genes were important in the early stages of cell transformation into malignancy, whereas genes such as *IL1RAP* and *IL1RN* were significant in the advanced stages.

Concerning the clinical studies available in databases, all genes in this study, except for *IL1RN*, which is more expressed in tumor tissue, showed higher expression levels in normal than in tumor tissue. This indicates the high level of inflammation that breast cancer generates in the adjacent tissue. In the tumor, the expression of these genes also affected the immune response in terms of innate immune cell infiltration, and this depended on the breast cancer subtype, and therefore ERα expression. However, the infiltrate of neutrophils was transversal and was found in all types of breast cancer with expression of the genes studied in this work, in contrast to dendritic cell and macrophage infiltration. The ERα expression was related to the genes studied and it was found that IL6ST gene expression was dependent on ERα expression. However, the gene expression of the CXCLs genes family has an inverse relationship to the expression of ERα, as shown by its negative correlation with the expression of the *ESR1* gene and its ER status negative. On the other hand, the results of the association between the *IL1R1*, *I1L1RN*, and *ILRAP* genes with ERα were less conclusive. Despite this, the importance of the expression of these genes in breast cancer is such that high *IL1R1* or *IL1RAP* is strongly related to patient survival.

Understanding the role of innate immune cells in carcinogenesis and progression can help improve therapeutic approaches targeting innate immune cells, increasing the likelihood of favorable prognosis predicated on inflammatory response and its potential to inform tailored therapeutic strategies. The results of this study may pave the way for novel treatment strategies considering now the target related to inflammation in breast cancer.

## Figures and Tables

**Table 1:** Raw data of genes selected for this study from the Affymetrix array U133A.

GeneBank	Gene Symbol	Ct/E	Ct/A3	E/A5	A3/A5	A5/T2	A3/T2
NM_000877	*IL1R1*	−1.1	4.5	2.9	−1.8	−2.3	−4.1
NM_002182	*IL1RAP*	2.1	1.1	−10.9	−5.4	8.6	1.6
AW0833557	*IL1RN*	−1.7	−18.1	−4.1	2.6	4.2	11.1
NM_002184	*IL6ST*	1.6	4.3	−1.7	−4.7	1.4	−3.4
NM_002090	*CXCL3*	2.4	4.6	1.7	−1.2	−51.9	−59.9
BG166705	*CXCL5*	4.6	2.7	−8.3	−4.8	−100	−100
NM_002993	*CXCL6*	2.8	−9.2	−4.2	6.1	−4.2	1.4

Ct/E: MCF-10F/estrogen; Ct/A3: MCF-10F/Alpha3; E/A5: estrogen/Alpha5; A3/A5: Alpha3/Alpha5; A5/T2: Alpha5/Tumor2; A3/T2: Alpha3/Tumor2.

**Table 2:** Phenotypical characteristics of an experimental breast cancer model: Alpha Model.

Cell lines	Agar ^a^	Invasion ^b^	Tumorigenicity ^c^	Immunocytochemistry ^d^			Classification ^e^
				ER	PgR	ErbB2	
MCF-10F	−	−	−	−	−	−	normal
MCF-10F + E	−	−	−	−	−	−	normal
A3	+	+	−	+	+	+	malignant
A5	+	+	+	+	+	+	tumorigenic
T2	+	+	+	+	+	+	tumorigenic

^a^ Agar: + signs indicate the capability of colony-formation, reference number [45,78]. ^b^ Invasion: Invasive characteristics of control MCF-10F and the cell lines after the various treatments that were scored 20 h after plating onto the Matrigel basement membrane. Invasiveness was determined using modified Boyden’s chambers constructed with multiwell cell culture plates and cell culture inserts. + sign means that cells crossed the filters. The experiments were repeated with three very similar passages, reference number [45]. ^c^ Tumorigenicity: + sign represents tumor formation in athymic mice. An average of six animals were used per group, reference number [45,78,79]. ^d^ Immunocytochemistry: + sign represents positive cells after staining, reference number [80]. ^e^ Classification: − sign means normal for every phenotypic characteristic. + sign means malignant for agar, invasion, and immunocytochemistry, but non-tumorigenic in the A3 cell line. Tumorigenic: + for a, b, c, and d phenotypical characteristics in the A5 and T2 cell lines. 17ß-Estradiol (E) was chronically used in the E and A5 cell lines.

## Data Availability

TIMER2.0 is freely available at http://timer.cistrome.org/ (accessed on 6 August 2023), reference number [46]. UCSC Xena online exploration tools are freely available at http://xena.ucsc.edu/ (accessed on 20 August 2023), reference number [47]. The data generated in the present study may be requested from the corresponding author.
